# Supplementary figures and images for: Lysine-Specific Demethylase 1 (LSD1) epigenetically controls osteoblast differentiation
Source: PLoS One. 2022 Mar 7;17(3):e0265027. doi: 10.1371/journal.pone.0265027 (PMC8901060; doi:10.1371/journal.pone.0265027)

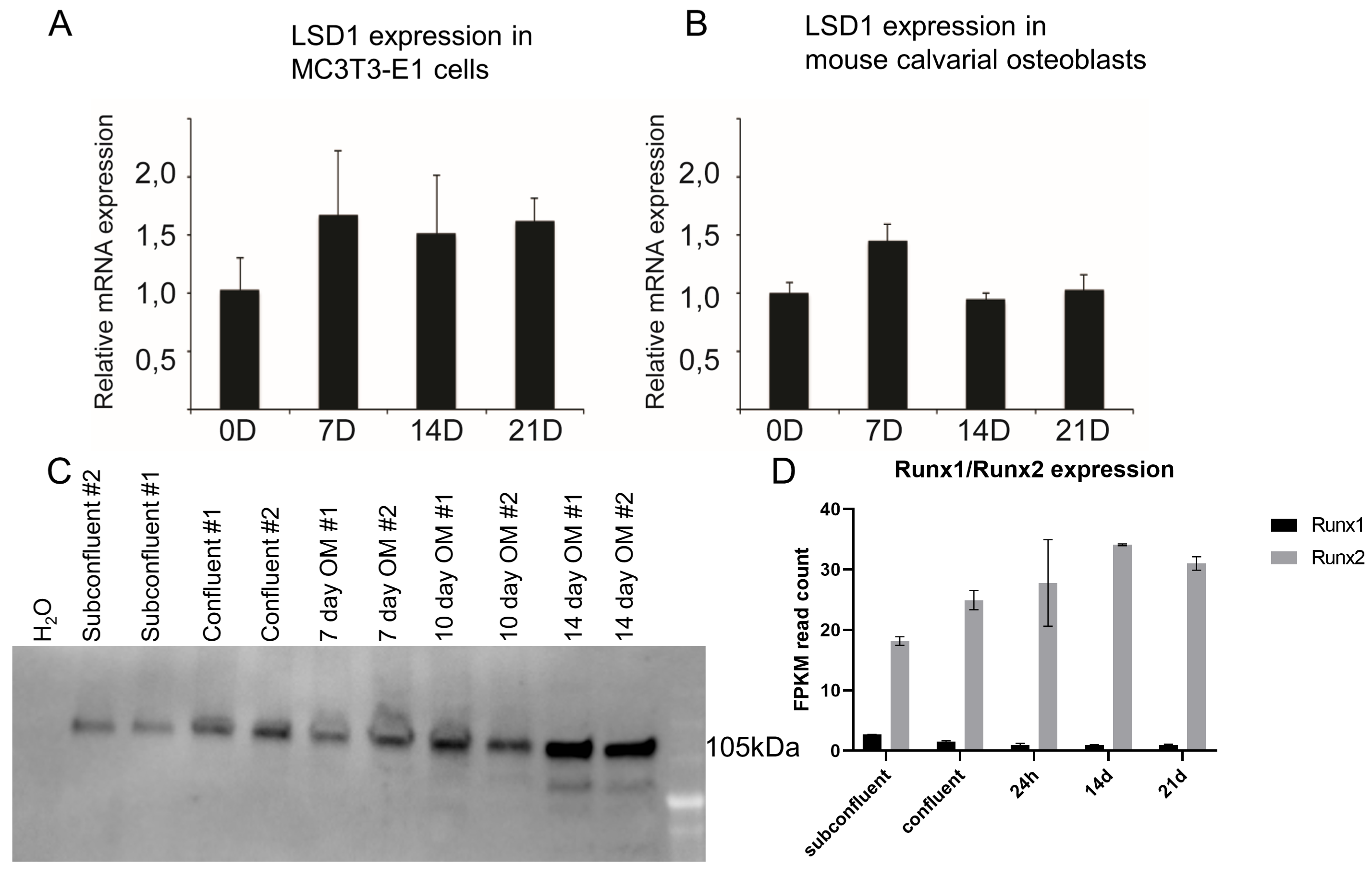

Supplement: S1 Fig — Lsd1 expression was stable throughout the differentiation culture of both MC3T3-E1 cells (A) as well as mouse calvarial osteoblasts (B) (n = 3). LSD1 protein expression was abundant and increased during osteoblast differentiation (C) in MC3T3-E1 cells. Both Runx1 and Runx2 were expressed throughout osteoblast differentiation, but Runx2 mRNA expression was clearly higher than Runx1 in the RNA-seq data (n = 2). (TIFF) [file pone.0265027.s001.tiff]

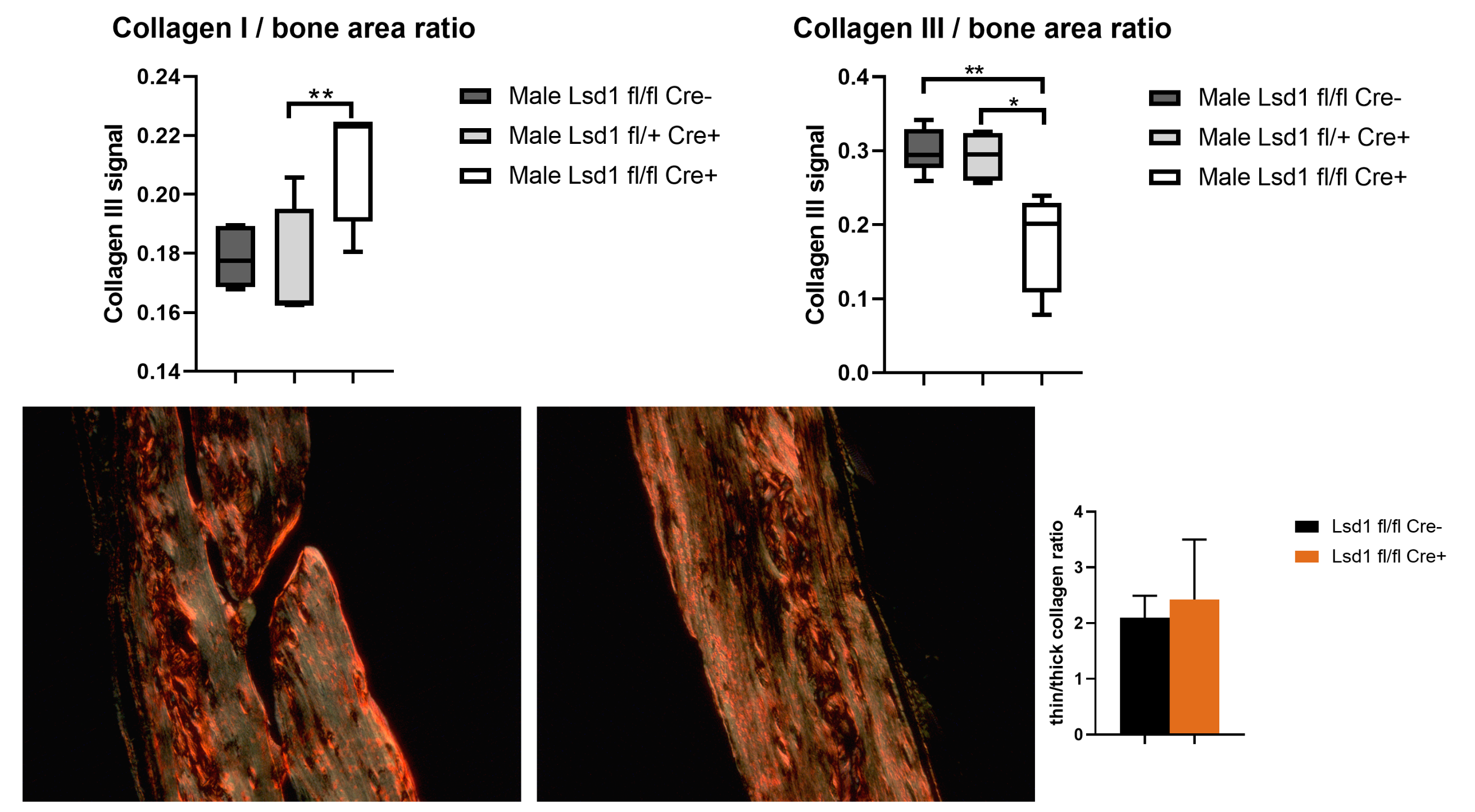

Supplement: S2 Fig — Histological analysis of the Picrosirius red stained tibial sections showed increased collagen I and decreased collagen III staining in the Lsd1Prrx1-/- mice compared to control when normalized to bone area, but the ratio of collagen I and III was unchanged (n = 4 per group). P-values for statistically significant differences are marked * P<0.05, ** P<0.01. (TIFF) [file pone.0265027.s002.tiff]

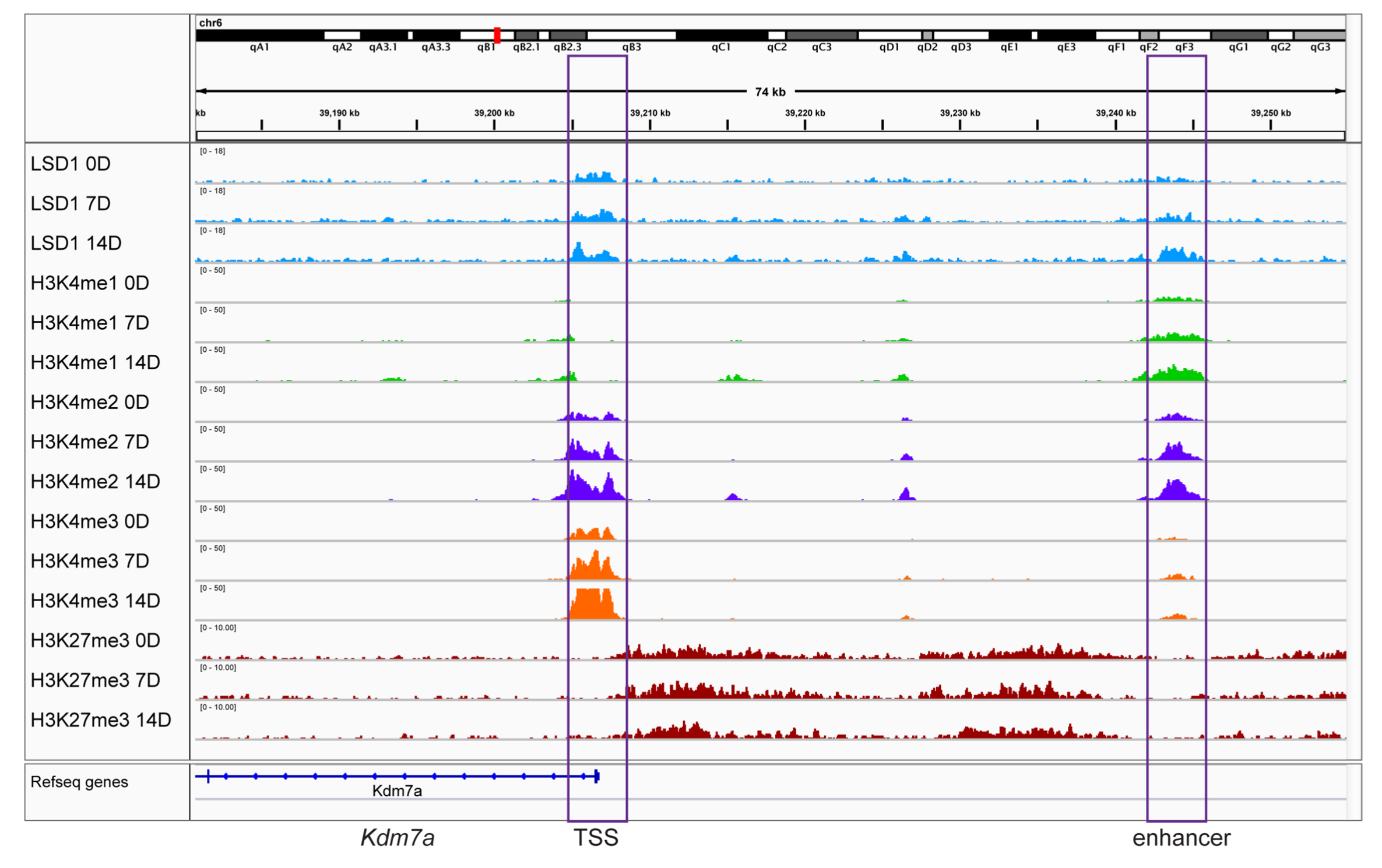

Supplement: S3 Fig — Kdm7a is an upregulated during differentiation and show Lsd1 binding both at proximal promoter and at upstream enhancer. (TIFF) [file pone.0265027.s003.tiff]

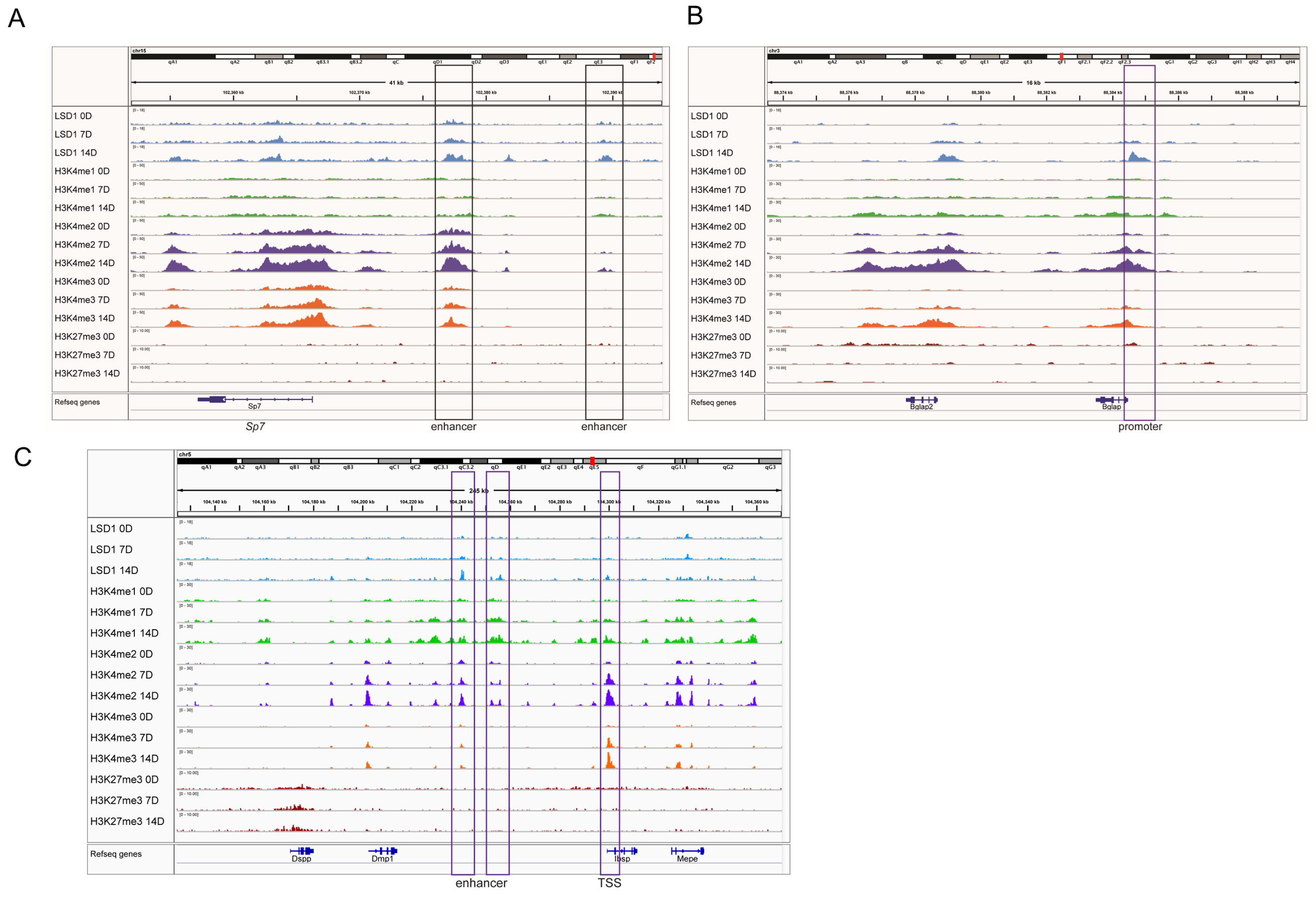

Supplement: S4 Fig — Osteogenic locuses for Sp7 (A), Bglap (B) and both Dmp1 and Ibsp (C) show correlation between high Lsd1, low H3K4me1 and high H4K4me2 at 14D. (TIFF) [file pone.0265027.s004.tiff]

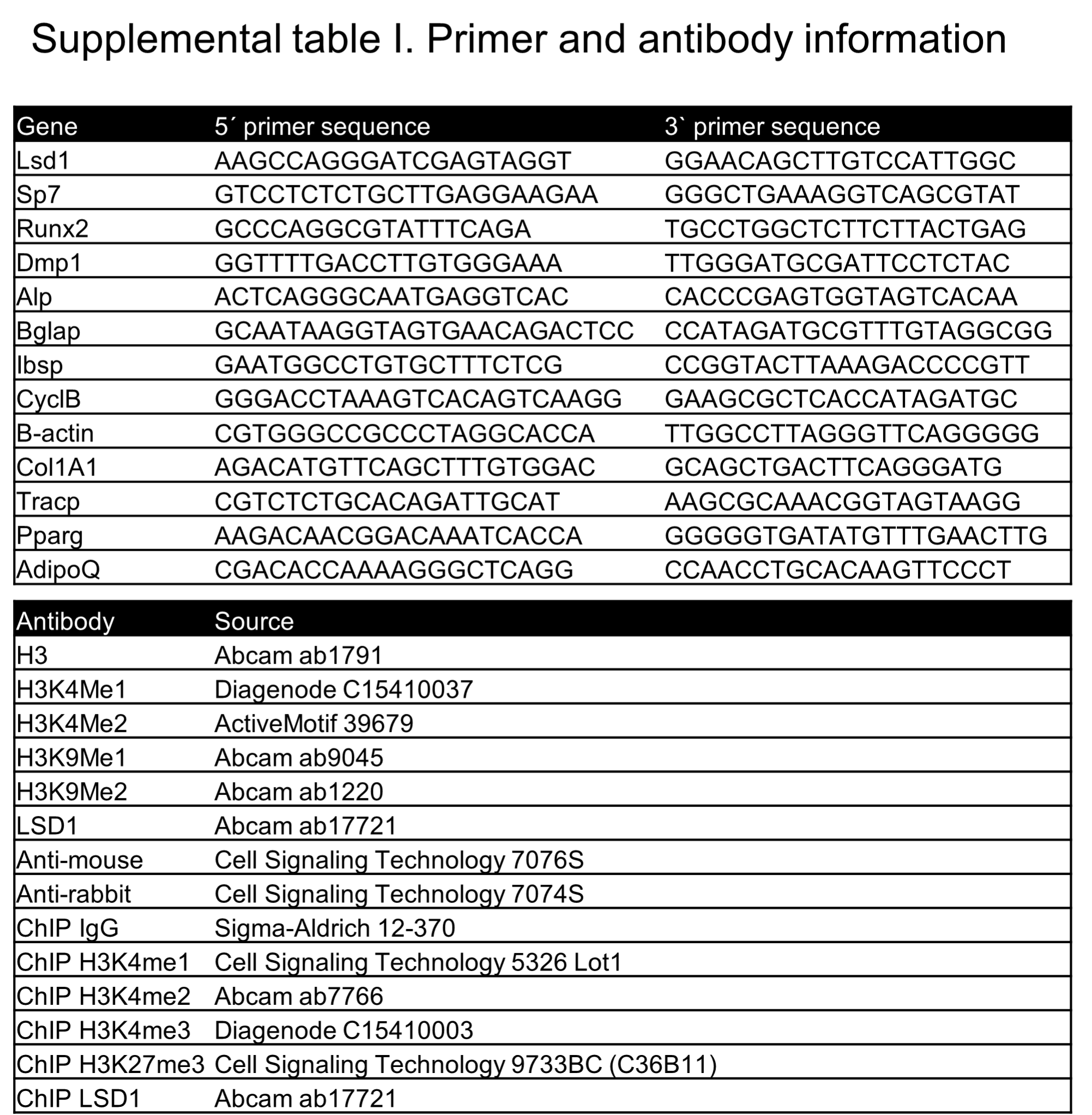

Supplement: S1 Table — (TIFF) [file pone.0265027.s005.tiff]
